# The relationship between internalised weight bias and biopsychosocial outcomes in children and youth: a systematic review

**DOI:** 10.1186/s40337-023-00959-w

**Published:** 2024-03-15

**Authors:** Tiarna Foster, Melissa Eaton, Yasmine Probst

**Affiliations:** School of Medical, Indigenous and Health Sciences, University of Wollongong, Wollongong, NSW Australia

**Keywords:** Internalised weight bias, Weight bias, Weight stigma, Internalised weight stigma, Youth, Eating disorders, Weight bias internalisation

## Abstract

**Objective:**

To synthesise the evidence on the relationships between internalised weight bias (IWB) and biopsychosocial health outcomes in individuals ≤ 25 years.

**Methods:**

A systematic review was conducted by searching five scientific databases up to May 2022 to retrieve studies that investigated associations between IWB and biopsychosocial outcomes. Articles with participants ≤ 25 years, at least one validated measure of IWB, one measure of a biopsychosocial outcome, and were observational were included. Excluded articles involved systematic literature reviews, case study reports, intervention studies, meta-analyses, grey literature, pilot, and feasibility studies. Quality assessment was carried out using the American Dietetic Association Quality Criteria Checklist. The protocol was registered with PROSPERO, ID number CRD42022323876.

**Results:**

Two hundred and sixty-six articles were identified. Nineteen were eligible for inclusion, (15 cross-sectional and 4 prospective). The Weight Bias Internalization Scale and the Weight Self-Stigma Questionnaire were the most used tools to assess IWB with large heterogeneity in tool types used to assess biopsychosocial measures. IWB had positive associations with psychopathology, eating disorder symptomology, higher BMI, being female, and experiences of weight stigma. It was negatively associated with quality of life, body image, physical activity, social ability, self-esteem, and socioeconomic status.

**Discussion:**

IWB associated with adverse biopsychosocial outcomes in children and youth populations. IWB may be more clinically relevant in assessing at-risk children and youth than physical weight due to its psychosocial aspects and ability to expand beyond the scope of BMI. Research would benefit from better assessment tools designed for children and youth that accurately measure IWB. Future research should focus on increased diversity and longitudinal study designs with children and youth-specific populations.

**Supplementary Information:**

The online version contains supplementary material available at 10.1186/s40337-023-00959-w.

## Abstract (plain language summary)

The objective of this systematic review was to  bring together the current evidence on the relationship between internalised weight bias (IWB) and health outcomes in individuals under the age of 25. The systematic review was conducted by searching five scientific databases to retrieve studies that investigated associations between IWB and biopsychosocial outcomes. Two hundred and sixty-six articles were identified, with nineteen eligible for inclusion into the review. IWB was associated with increased mental illness, eating disorder symptoms, BMI, being female, and experiences of weight stigma. It was associated with a decreased quality of life, body image, physical activity, social ability, self-esteem, and socioeconomic status. Overall IWB was found to be associated with negative health and social outcomes in children and youth populations. Future research in this area should focus on increased diversity, longitudinal study designs and designing  children and youth specific tools that accurately measure IWB.

**Supplementary Information:**

The online version contains supplementary material available at 10.1186/s40337-023-00959-w.

## Public significance statement

Results from this study revealed that significant associations exist between internalised weight bias and negative biopsychosocial outcomes in a population below 25 years of age. The results add to the growing body of literature, with findings helping to inform health care professionals of the importance of screening for internalised weight bias and recognising its signs in children and youth. It may also provide guidance when developing individual and population level nutrition interventions and educational programs.

## Introduction

Weight bias is a form of prejudice that leads to stigmatisation, social rejection and the devaluation of individuals who do not comply with social norms for weight [1, 2]. This can transform into internalised weight bias (IWB) when individuals become aware of, and internalise these conceived notions, proceeding to mistreat or devalue themselves due to a perceived self-classification of overweight (OW) [3]. Research related to IWB is gaining attention due to evidence showing an association with negative health outcomes socially, psychologically, and physically. A study by Puhl et al. reported that 44% of Americans experience IWB [4] with presently no prevalence data available for Australians.

Studies have demonstrated consistent links between IWB and adverse psychological and physical health outcomes within adults [5–7]. IWB has been identified, through cross-sectional studies, to increase adverse mental health indices such as depression, anxiety, poor body image, health-related quality of life, and eating disorder symptomology, even when controlling for BMI, with it playing a key role in psychosocial health [8, 9]. Though there are fewer studies examining physical health, associations have been observed between IWB and a higher BMI, lower self-efficacy for engaging in health-promoting behaviours, and poor dietary adherence in treatment-seeking populations [10].

IWB does not exist separate to external weight stigma, but rather is a product of it and the immediate and wider social environment [4]. Lived experiences of external weight stigma occur through our social networks and contribute to the multifactorial nature of IWB. Experiences from the home environment, and peers, such as weight-based teasing have all been reported to contribute to the development of IWB [11], with children and youth reporting higher levels of weight based teasing and vulnerability [12].

The influence of a child’s environment in addition to their life experiences are vital to consider when addressing any form of stigma. The prevalence of eating disorders is highest between the ages of 12 and 25 years, though weight bias and dieting behaviours may appear as young as 5 years of age [13, 14]. Children are more dependent on their environment for social feedback making them vulnerable to the negative consequences of IWB [15, 16]. This period of development is one in which a self-depreciating mindset and perception of oneself are is presumed to appear with long-term impacts and beliefs likely to track into adulthood [17, 18]. Angel. et al., demonstrated that children and youth who utilised extreme weight control behaviours in early adolescence demonstrated an 8.4–20.4% increase in these behaviours as they entered young adulthood [18], highlighting the need for early intervention. Through greater research and early intervention, health professionals can aid in preventing the chronic nature of these developed beliefs and address them before they compound into adulthood [19].

Research to date within populations under and over 25 largely investigates IWB in treatment seeking populations, with research showing that these populations are at a higher risk for scoring lower on health related quality of life tests when compared to peers within the community in regard to IWB [20, 21]. This has then impacted on their treatment/health outcomes. A study assessing IWB scores with a “lean” population (mean: 22.28 ± 1.89 kg/m^2^, range 15.80–24.98 kg/m^2^, using the classification of ‘normal weight’ or ‘underweight’ (BMI < 25 kg/m^2^) according to the National Institutes of Health's weight classification guideline) found that 38.6% of respondents self-reported as ‘overweight’ or ‘obese’ [22]. They proceeded to find that respondents who believed themselves to be ‘OW/obese’ had significantly greater IWB scores than those who accurately perceived themselves as ‘normal weight’. In addition, formerly ‘overweight’ persons may continue to internalise weight bias even after weight loss [23], hence the importance of continued monitoring for IWB across the weight spectrum.

**Fig. 1:**
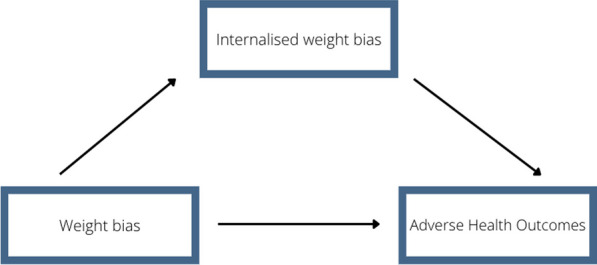
Mediational relationship between weight bias, internalised weight bias, and adverse health outcomes

Further, to better understand the relationship between health, experiences of weight stigma and weight, it is important that we understand its mediators. IWB demonstrates a mediational relationship [24, 25] between weight bias and adverse health outcomes (Fig. 1), with evidence highlighting a potentially damaging impact on individuals regardless of BMI and experiences of stigma. This highlights a need to look beyond physical weight when addressing IWB and address biopsychosocial outcomes as they demonstrate a reciprocal predicative relationship with IWB; whereby increasing IWB increases adverse outcomes and vice versa [26]. For example, an increase in IWB may lead to decreased self-efficacy and motivation to engage in health-promoting and social behaviours, which may in turn negatively influence social and physical outcomes [27].

Health outcomes are vital to explore within child and youth with early experiences creating biological ‘memories’, that shape development, and set the foundations for lifelong health [28]. Psychosocial problems in children can lead to serious consequences for social competence, learning, and lifelong physical health outcomes. Distinguishing between children and adolescents is also of importance with clear cognitive, physical, and psychosocial differences occurring between the age groups due to increased vulnerability related to hormonal changes, changes in appearance, and transition toward adult roles and responsibilities [29]. Changes associated with puberty are a required consideration when assessing children and youth’s self-image and worth due to increased vulnerability in this population. This population is vital to investigate independent to adult populations as children and youth differ cognitively, physically, and psychosocially, with results unable to be generalised. Many lifelong adverse health behaviours are believed to originate in the earlier years before compounding into adulthood, hence a need for greater research to allow for early intervention [17–19].

This systematic review aimed to synthesise and evaluate the evidence on the relationship between IWB and biopsychosocial health outcomes of individuals’ ≤ 25 years of age to identify associations and limitations within the literature. It aims to highlight key research gaps that can inform future studies around IWB. The findings may help to inform health care professionals of the importance of screening for IWB and recognising its signs in child and youth populations.

## Methods

### Search strategy

A systematic literature search was conducted to identify studies that investigated an association between IWB and any biopsychosocial outcomes (e.g. BMI, depression, eating disorder symptomology) in individuals’ ≤ 25 years old. This systematic review followed the Preferred Reporting Items for Systematic Reviews and Meta-Analyses (PRISMA) and the Cochrane handbook for systematic review guidelines and was reported according to the PRISMA 2020 checklist [30] (Fig. 1). The protocol for this search is registered with PROSPERO, ID number CRD42022323876.

The literature search was conducted April–May 2022 using five health-related scientific databases: PsycInfo, Web of Science, Cinahl, Medline (EBSCOhost) and Scopus. Database and keyword selection was informed by previous studies of IWB and through pilot searches. A preliminary search of the literature was used to inform the choice of key words and MeSH terms. The final search comprised the following keyword combinations: child,*adolescen,* teenager,* youth, “young person,” “under 18,” “under eighteen,” “school aged,” “university student,* “preschool children,” “under 25,” “under twenty-five,” intervention,* “trial,” rct, observation,* cohort, longitudinal, cross-sectional, prospective, retrospective, quasi-experimental, “weight bias” “weight-bias,” “weight discrimination,” “weight based discrimination,” “weight-based discrimination,” “weight prejudice,” “weight stigma,” “weight-stigma,” “weight bias internalization,” “weight bias internalisation.” Keywords and boolean combinations were applied to each of the databases and adapted to suit database specific needs only.

### Eligibility criteria

Articles published in peer-reviewed journals with participants ≤ 25 years that included at least one validated measure of internalised or self-directed weight bias (e.g. Weight Bias Internalization Scale or Weight Self-Stigma Questionnaire), in addition to at least one biopsychosocial outcome variable (e.g. diagnosed or self-reported anxiety or depression, self-esteem, disordered eating, body weight, BMI, physical activity etc.) were included. Only observational quantitative study designs (e.g., cross-sectional, longitudinal) were included and no limitations were placed on the year of publication. The term youth was used to include both adolescents and youth, and encompasses the ages 13–25 years. Children was used to refer to participants ≤ 12 years. In incidences when both youth and children are referred to, the phrase a population under the age of 25 was utilised. There was no pre-determined lower limit for inclusion of age.

Exclusion of studies was informed for the Cochrane handbook and previous studies [10]. Excluded studies included systematic review and meta-analyses studies, case study reports, intervention studies and meta-analyses, grey literature, pilot and feasibility studies. Reference lists of these articles were manually searched and cross-checked against eligibility criteria to ensure eligible papers had not been missed.

### Article selection

The search outcomes were imported to Covidence for duplicate removal prior to title and abstract screening against the eligibility criteria by one reviewer (TF). Full text screening was completed by three reviewers (TF, YP, ME) to exclude studies that did not include a measure of internalised or self-directed weight bias and a biopsychosocial measure. The proportionate agreement was 63% and 59% between reviewers, with all studies reviewed independently by two researchers before being included in the review.

### Data abstraction and synthesis

The following data was extracted from all included reports: country where data was collected, publication year, author/s, study design, population information (e.g., size, source, etc.), sample demographics (e.g., age, gender, BMI, % BMI category if available, income, parental education level, and ethnicity when available etc.), measures used to assess internalised weight stigma and measures used to assess a biopsychosocial outcomes and outcome data. Studies were divided into three categories: (1) psychological outcomes; (2) physical outcomes; and 3) social outcomes.

Where articles reported multiple biopsychosocial outcomes, articles were not categorised as one single outcome but rather each outcome was individually categorised to each category. Therefore, the total number of outcomes may exceed the total number of included studies and studies may appear across multiple categories. Finally, for instances where multiple outcomes followed the same theme they were categorised under the same definition. For example, all eating disorder symptomology outcomes were categorised under one category due to the heterogeneity of the data.

### Quality assessment

Publication bias was assessed by one researcher (TF) using the American Dietetic Association Evidence Analysis Library Quality Criteria Checklist [31]. As per Cochrane guidelines, quality assessment was conducted following inclusion of studies to allow for inclusion to be guided by the inclusion criteria and avoid influence. This provided insight into various biases related to each of the studies.

Due to the limited research designs (e.g., longitudinal or experimental), criteria were modified based on prior research on IWB. Quality assessment of studies included in this review addressed: cross-sectional versus longitudinal study design; validity of biopsychosocial outcomes measure: self-report versus objective measurement; and validity of the IWB measure.

## Results

**Fig. 2:**
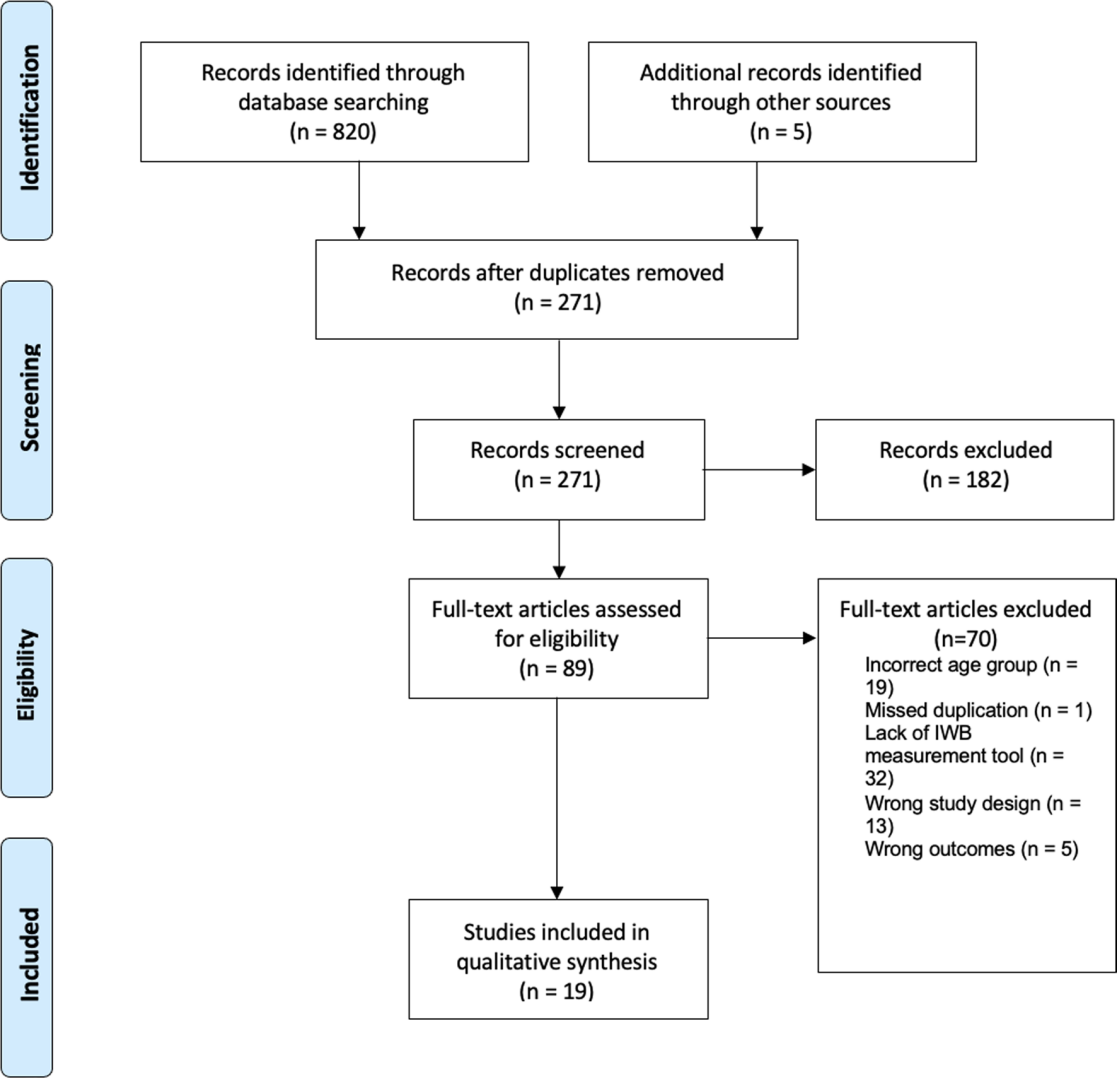
PRISMA flowchart of article screening and selection process

Figure 2 depicts the selection flow as per PRISMA guidelines. Across the databases, after duplicates were removed, 266 articles were identified. Six additional articles were identified during manual screening of reference lists. Following quality assessment, 19 studies remained for data extraction.

### Characteristics of included studies and participants

Of the 19 studies included, 21% (n = 4) were prospective studies and 79% (n = 15) were cross-sectional. Fifteen relied on retrospective data analyses. Ten studies were included from Eurocentric countries (Germany [21, 32–34], Australia [24], USA [35–38], Canada [32]), six from Asian (China [26, 39], Hong-Kong [26], Korea [40]) and three from Middle Eastern countries (Iran [41, 42], UAE [43]). A majority of studies (68.4%) were published between 2019 and 2021. The earliest study included was published in 2012.

Only six studies provided information on the ethnicity of participants. A majority (69.83%) of identified ethnicities were white. Only, 14.53% were identified as black, 10.76% as Hispanic or Latino, 8.29% as Asian, and 5.80% were identified and classified as other by the study authors. Of the participants, 61.45% were female, and 38.55% were male, with four studies exclusively investigating a female population. Three studies [21, 32, 33] investigated the role that sex played on IWB scores and found females to have higher IWB scores, whilst two found no difference [35, 38]. Lastly, the ages of participants ranged from 5 to 25 years. Three studies involved dyads of one parent and child [39].

### Tools used to measure internalised weight bias

To assess IWB, studies predominantly used the Weight Bias Internalization Scale (WBIS; *n* = 16, 84.21%) or a modified version and the Weight Self-Stigma Questionnaire (WSSQ; *n* = 5, 26.32%). One study that utilised the Weight- and Body-Related Shame and Guilt Scale (WEB-SQ) was included in this review due to it explicitly stating that it was used as a measure of IWB. Seven of the 19 studies modified the Weight Bias Internalization Scale: four used a modified WBIS (WBIS-M), two a child WBIS (WBIS-C), one a WBIS tool for youth (WBIS-Y) [26, 32, 44], Table 1.

**Table 1:** Summary of included studies with outcomes categorised as psychological, physical and social factors

Reference	Study design (country)	n (% females)	Mean age, years (range)	Ethnicity	Reported BMI, kg/m^2^	Measure of IWB	IWS mean	Relationship		
								Psychological	Physical	Social
Ahorsu et al. [41]	Prospective (Iran)	1497 (54.30%)	15.10 ± 6.00		baseline = 31.8 ± 5.4 six-months = 33.8 ± 5.1	WBIS	3.8 (± 0.97)	**IWS versus binge eating at follow-up: r = 0.22, *p* = < 0.01 IWS versus psychological distress at follow-up: r = 0.15, *p* = < 0.01 IWS versus food addiction follow-up: r = 0.21, *p* = < 0.01		
Ciupitu-Plath et al. [21]	Cross-sectional (Germany)	191 (51.31%)	(13–19)	German: 46.6% Other: 49.74%	BMI 26–29 kg/m^2^ = 15.71% BMI > 30 kg/m^2^ = 33.51% BMI > 35 kg/m^2^ = 47.74%	WBIS-Y	Girls: 4.01 Boys: 3.54	*Self-esteem: α = − 0.70 Internal related LoC: α = − 0.01 External related LoC: α = 0.28 Self-efficacy: α = − 0.39 generic health-related quality of life: α = − 0.50	*BMI-SDS: α = 0.20 obesity-specific QoL: α = − 0.77	
Fan et al. [39]	Cross-sectional (China)	430 dyads (44%)	10.07 ± 1.42; (8–12)		18.47 ± 4.16	WBIS; WSSQ	WBI: 1.59 (0.56) WBI Boys:1.50 (0.53) BMI > 25 kg/m^2^: 1.47 (0.48) BMI ≤ 25 kg/m^2^: 2.06 (0.62)	**Perceived weight stigma: 0.81, *p* < 0.001	**Sex: r = 0.06 BMI: r = 0.025, *p* < 0.001	**Weight stigma: r = 0.39, *p* < 0.001
Gmeiner et al. [32]	Prospective (Germany)	1463 (51.74%)	8.35, ± 0.94; (6–11)		BMI < 18.5 kg/m^2^ = 6.08% BMI 18.5–25 kg/m^2^ = 80.72% BMI > 25 kg/m^2^ = 3.19%	WBIS-C	Girls: 1.68 (0.62) Boys:1.56 (0.56) BMI > 25 kg/m^2^: 2.16 (0.68) BMI ≤ 25 kg/m^2^: 1.53 (0.52)	***Body dissatisfaction: r = 0.36, *p* < 0.001 Relevance of one’s own figure: r = 0.2, *p* < 0.001 Self-esteem: r = − 0.05, *p* = 0.046 Depressive symptoms r = 0.24, *p* < 0.001	***BMI-SDS: r = 0.43, *p* < 0.001	***Weight-related teasing: r = 0.28, *p* < 0.001
Gmeiner et al. [34]	Cross-sectional (Germany)	1061 (52.1%)	11 ± 0.9; (9–13)		BMI < 18.5 kg/m^2^ t: 8% BMI 18.5–25 kg/m^2^: 78.3% BMI 25–30 kg/m^2^: 8.1% BMI > 30 kg/m^2^: 5.6%	WBIS-C	1.55 ± 0.55) BMI > 25 kg/m^2^: 2.09, ± 0.66 BMI ≤ 25 kg/m^2^: 1.46 ± 0.47	¶¶¶ WBIS-C versus SDQ: AUC = 0.67, *p* < 0.001 WBIS-C versus SCOFF: AUC = 0.77, *p* < 0.001		
Himmelstein et al. [38]	Cross-sectional (USA)	148 (50%)	15.97 ± 1.25	White: 90.6% Black: 2.0% Asian: 2.7% Latino: 4.7%	27.06 ± 4.39	WBIS-M	5.45 (0.88)	***WBI versus responding to bullying with negative emotions: β = 0.62, *p* = < 0.001 WBI versus responding to bullying with indifference: β = − 0.07, *p* = 0.487 WBI versus coping with avoidance: β = 0.39, *p* = < 0.001 WBI versus coping with eating: β = 0.70, *p* = < 0.001		
Kamolthip et al. [26]	Cross-sectional (Hong-Kong)	437 dyads (43.90%)	10.07 ± 1.43; (8–12)		18.59 ± 4.25	WBIS; WSSQ	Ative group: r = 22.51 (8.04) Non-active group: r = 24.10 (8.62) WSSQ active group: r = 22.67 (8.58) WSSQ non-active group: r = 24.34 (9.36) Self-stigma active group: r = 34.54 (11.67) Self-stigma non-active group: r = 37.13 (12.67)	**Physical activity versus self-stigma r = − 0.11, *p* = .029 Self-stigma versus Kid-KINDL child-rated: r = − 0.38, *p* < .001 Self-stigma versus parent-rated r = − 0.21, *p* < .001 Self-stigma versus SMU r = − 0.60, *p* < .001 Self-stigma versus STU r = − 0.34, *p* < .001		
Lin et al. [42]	Cross-sectional (Iran)	934 (52.46%)	15.7 ± 1.2; (13–18)		33.0 ± 4.7	WBIS	3.0 ± 0.5	¶ DASS-21: r = 0.38 (*p* < 0.001)	¶ zBMI: r = 0.23 (*p* < 0.001)	
Maïano et al. [45]	Cross-sectional (Canada)	156 (48%)	16.31 ± 0.85 (14–19)		BMI 26–29 kg/m^2^ (76.3%) BMI ≥ 30 kg/m^2^ (23.7%)	WWSQ	Self-devaluation: 0.778 fear of enacted stigma: 0.828	**WSSQ (self-devaluation) versus Self-esteem: r = − 0.437, *p* < 0.001 WSSQ (self-devaluation) versus Physical appearance: r = − 0.428, *p* < 0.001 WSSQ (self-devaluation) versus Anxiety: r = 0.242, *p* < 0.01 WSSQ (self-devaluation) versus Depression: r = 0.257 *p* < 0.01 WSSQ (self-devaluation) versus FNAES: r = 0.429, *p* < 0.001 WSSQ (self-devaluation) versus Eating-related control: r = 0.241 *p* < 0.01 WSSQ (self-devaluation) versus Vomiting-purging behaviours: r = 0.140, *p* < 0.05 WSSQ (self-devaluation) versus Food preoccupation: r = 0.366, *p* < 0.001 WSSQ (self-devaluation) versus Eating-related guilty: r = 0.429, *p* < 0.001		
O'Brien et al. [24]	Cross-sectional (Australia)	634 (73.40%)	19.70 ± 3.07	White: 60% Asian or pacific islander: 37% Black: 3%	22.40 ± 4.14	WBIS-M	BMI > 25 kg/m^2^: 3.91 (1.65) BMI ≤ 25 kg/m^2^: 2.77 (1.29)	**WBIS versus BMI: r = 0.15, *p* < 0.001 WBIS versus Gender: r = 0.23, *p* < 0.0001 WBIS versus Stigma total: r = 0.56, *p* < 0.0001 WBIS versus DASS-21: r = 0.51, *p* < 0.0001 WBIS versus Emotional eating: r = 0.52, *p* < 0.0001 WBIS versus Uncontrolled eating: r = 0.47, *p* < 0.0001 WBIS versus LOCES-B: r = 0.65, *p* < 0.0001		
O'Hara et al. [43]	Cross-sectional (UAE)	420 (100%)	23.12 ± 4.62		24.09 ± 5.77	WEB-SG	20.15 (11.65)	**WEB-SG versus BMI: r = 0.47** WEB-SG versus EAT-26: r = 0.43** WEB-SG versus TF: r = 0.30** WEB-SG versus BT: r = 0.39** WEB-SG versus AFA: r = 0.43** WEB-SG versus SAAS: r = 0.56** WEB-SG versus SATAQ: r = 0.35** *p < 0.055; **p < 0.01		
Pakpour et al. [46]	Cross-sectional (China)	287 (46.70%)	10.21 ± 1.31 (8–12)		BMI ≤ 25 kg/m^2^: 66.2% BMI > 25 kg/m^2^: 33.8%	WSSQ; WBIS	191 ± 0.75 WBIS: 2.28 ± 0.64	**WBIS versus PWD: r = 0.35, *p* < 0.001 WBIS versus Kid-KINDL: r = − 0.32, *p* < 0.001 WBIS versus Sizing me up: r = − 0.51, *p* < 0.001 WSSQ versus PWD: r = 0.38, *p* < 0.001WSSQ versus Kid-KINDL: r = − 0.37, *p* < 0.001 WSSQ versus Sizing me up: r = − 0.59, *p* < 0.001		
Pik Chu Wong et al. [44]	Cross-sectional (Hong-Kong)	124 dyads (40%) OW: 9.36 ± 1.17; 8–12 Non-OW = 9.73 ± 1.28	(8–12)		BMI > 25 kg/m^2^ = 22.86 ± 2.32 BMI ≤ 25 kg/m^2^ = 16.27 ± 2.10	WBIS; WSSQ	BMI ≤ 25 kg/m^2^ group: 26.49 ± 8.68 BMI > 25 kg/m^2^ group 21.58 ± 7.54 WSSQ BMI ≤ 25 kg/m^2^ 14.50 ± 4.89 WSSQ BMI > 25 kg/m^2^ 11.02 ± 4.37	**OW group WBIS versus BSRS-5: r = 0.525, *p* < 0.001 WSSQ versus BSRS-5: r = 0.336, *p* < 0.001 Non-OW group WBIS versus BSRS-5: r = 0.376, *p* < 0.001 WSSQ versus BSRS-5: r = 0.298, *p* < 0.001		
Puhl et al. [35]	Cross-sectional (USA)	15.26 ± 1.63	(13–18)	White: 90.5% Black: 2% Asian: 2.7% Latino: 4.7%	27.06 ± 4.39	WBIS	5.45 ± 0.88 Girls: 5.40 ± 0.97 Boys: 5.49 ± 0.78 BMI ≤ 25 kg/m^2^: 5.02* ± 0.59 BMI 26–29 kg/m^2^: 5.40* ± 0.87 BMI > 30 kg/m^2^: 5.85* ± 0.92	***WBI versus Binge eating: β = − 0.18 WBI versus Eating to cope: β = 0.50 WBI versus Female: β = 0.04 WBI versus BMI; β = 0.02 WBI versus Race: β = − 0.05 WBI versus Frequency of mother’s comments about adolescents: β = 0.24 WBI versus Frequency of mother’s comments about their own weight: β = 0.20		
Ra et al. [40]	Cross-sectional (Korea)	233 (100%)	13.43 ± 0.68; (12–14)		53.92 ± 30.30	WBIS	0.08 ± 1.01	***Fear of obesity: β = .43, *p* < .001		Attachment to teacher: β = − .11, *p* = .029 Perceived socio-cultural pressure β = .34, *p* < .001
Roberto et al. [36]	Cross-sectional (USA)	57 (80.70%)	15.65 ± 1.08; (14–18)	White: 50.9% Hispanic: 31.6% Hispanic African American: 12.3% Other: 5.3%	46.92 ± 7.86 (35.90–72.30)	WBIS	4.29 ± 1.52	**WBIS versus age: r = 0.169 WBIS versus Beck Depression Inventory: r = 0.520, *p* < 0.01 WBIS versus Youth Self-Report Total: r = 0.310, *p* < 0.05 WBIS versus Paediatric Quality of Life Teen Self-report: r =—0.483, *p* < 0.01 WBIS versus Multidimensional Anxiety Scale for Children: r = 0.467, *p* < 0.01 WBIS versus Eating Concern Subscale r = 0.581, *p* < 0.01 WBIS versus Restraint subscale: r = − 0.081 WBIS versus Shape Concern Subscale: r = 0.813, *p* < 0.01 WBIS versus Weight Concern Subscale: r = 0.570, *p* < 0.01 WBIS versus BMI: r = 0.044		
Romano et al. [37]	Cross-sectional (USA)	Sample 1; 1228 (75.8%) 22.27 ± 5.83 Sample 2 = 1368 (75.80%)	20.60 ± 3.47	White: 41.2% Black or African American: 37.8% Multiracial:12.7% Asian, Asian American, Native Hawaiian, or Pacific Islander: 5.0% Other race: 2.7% American Indian or Alaskan Native: 0.6% Hispanic or Latinx: 14.4%	26.47 ± 6.57	WBIS-M	BMI ≤ 25 kg/m^2^: 2.59 (1.27) BMI 26–29 kg/m^2^ group mean: 3.39 (1.48) BMI > 30 kg/m^2^ group mean: 4.07 (1.57)	¶¶ WBI versus BMI r = 0.385, *p* < 0.001 WBI versus Experienced weight stigma: r = 0.440, *p* < 0.001 WBI versus Body dissatisfaction: r = 0.740, *p* < 0.001 WBI versus Binge eating: r = 0.439, *p* < 0.001 WBI versus Purging: r = 0.377, *p* < 0.001 WBI versus Restricting: r = 0.206, *p* < 0.001 WBI versus Excessive exercise: 0.095, *p* < 0.01 WBI versus Muscle building: 0.055 (not sig)		
Saffari et al. [47]	Cross-sectional (China)	391 (100%)	22.85 ± 4.03		21.30 ± 3.53	WSSQ	30.49 (10.58) High physical activity group: 30.40 (10.01) Moderate physical activity group: 26.37 (10.82) Low physical activity group: 31.74 (10.33)	¶¶ WRSS versus Age: r = 0.04 (0.40) WRSS versus Weight status: r = 0.40 (< 0.001) WRSS versus Psychological distress: r = 0.45 (< 0.001)		
Zub et al. [33]	Prospective (Germany)	1047 (52%)	Time 1: 9.0 ± 0.9; (7–11) Time 2: 10.5 ± 0.97; (7–11)		BMI <18.5 kg/m^2^ = 6.3% BMI 18.5–25 kg/m^2^ = 81.4% BMI > 25 kg/m^2^ = 12.4%	WBIS-C	WBIS:23.33 (8.33) WSSQ Q1-6 score: 12.51 (4.70) WSSQ Q7-12 score: 10.94 (4.88)	**Perceived Weight stigma: r = 0.81 Child related KID-KINDL: r = − 0.38	**BMI: r = 0.25	**Experienced weight stigma: r = 0.39 Sizing me up: r = − 0.60 Sizing them up: r = − 0.34

*AFA* Anti-Fat Attitudes Questionnaire, *IPPA-R* Assessment of Attachment with Parents during Peer Attachment-Revised, *BDI* Beck Depression Inventory, *BISS* Body Image States Scale, *CBC* Child Behaviour Checklist, *CHQ* Child Health Questionaries, *DASS-21* Depression, Anxiety, Stress Scale-21, *DEQB-K* Dutch Eating Behaviour Questionnaire for Children, *EAT* Eating Attitudes and Teens, *EAT-26* Eating Attitudes Test 26-item version, *EDE-Q* Eating Disorder Examination Questionnaire, *EPSI* Eating Pathology Symptoms Inventory, *EDDS* Everyday Discrimination Scale, *EWS* Experienced Weight Stigma, *FNAES* Fear of Negative Appearance Evaluation Scale, *DTGA* German Depression Test for Children, *KAT* German Anxiety Test for Children, *HADS* Hospital Anxiety and Depression Scale, *LOCES* Loss of Control of Eating Scale, *MASC* Multidimensional Anxiety Scale for Children, *PEDS-QL* Paediatric Quality of Life Inventory 4.0, *POTS* Perception of Teasing Scale, *PANAS* Positive and Negative Affect Schedule, *RSE* Rosenberg Self-Esteem Scale**,** *SDQ-II* Self-Description Questionnaire II**,** *SMU* Sizing Me UP (child-rated report) and *STU* Sizing Them Up (parent-rated report),), *SABAS* Smartphone Application-Based Addiction Scale, *SAAS* Social Appearance Anxiety Scale, *SATAQ-ED* Sociocultural Attitudes Toward Appearance Questionnaire for people with Eating Disorders, *SSI-B* Stigmatizing Situations Inventory—brief, *SDQ* Strengths and Difficulties Questionnaire, *TFEQ-R18* Three Factor Eating Questionnaire, *YFAS-CKid-KINDL* Yale Food Addiction Scale for Children, *YSR* Youth Self-Report, *WSS* Weight Self Stigma

*Crohnbach, **Pearsons correlation, ***Regression, ¶Mediation, ¶¶Bivariate correlation, ¶¶¶ROC analysis

Table 1 summarises all included studies on IWB and outcomes in children and youth. All studies included at least one validated measure of a biopsychosocial outcome, with high variability in the types of tools used.

### Psychological outcomes

**Fig. 3:**
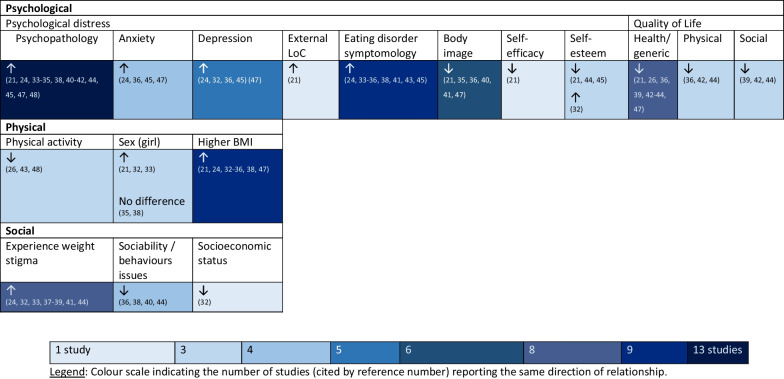
Heat map indicating the relationships reported in included studies of IWB. Arrows indicate the direction of relationship with IWB as scores increase. Abbreviations: *BMI* Body Mass Index, *IWB* Internalised weight bias, *LoC* Locus of control. Legend: Colour scale indicates the number of studies (cited by reference number) reporting the same direction of relationship

All nineteen studies found positive associations between higher levels of IWB and at least one negative psychological outcome [6–28, 31, 34–36, 38–40]. The findings are summarised in Fig. 3 which demonstrates the associations between IWB and psychological distress, eating disorder symptomology, self-esteem, and quality of life as IWB scores increased.

Positive associations were seen between prevalence of eating disorder symptomology (binge eating, eating to cope, eating restriction, bulimia, oral control, restrained eating, uncontrolled eating, emotional eating), and pathopsychological (emotional problems, depression, anxiety, psychological distress) as IWB scores increased.

IWB was significantly and positively associated with elevated levels of psychopathology and negative psychosocial outcomes [33, 36, 41, 45] across sex and weight groups [33, 41]. IWB mediated the relationship between elevated BMI and quality of life [39], and psychosocial problems [21, 33, 48], with IWB shown to be more important than weight status in explaining psychological functioning [33] via cross-sectional studies. Five studies found IWB significantly and negatively correlated with health-related quality of life [21, 26, 43, 44].

### Physical outcomes

**Fig. 4:**
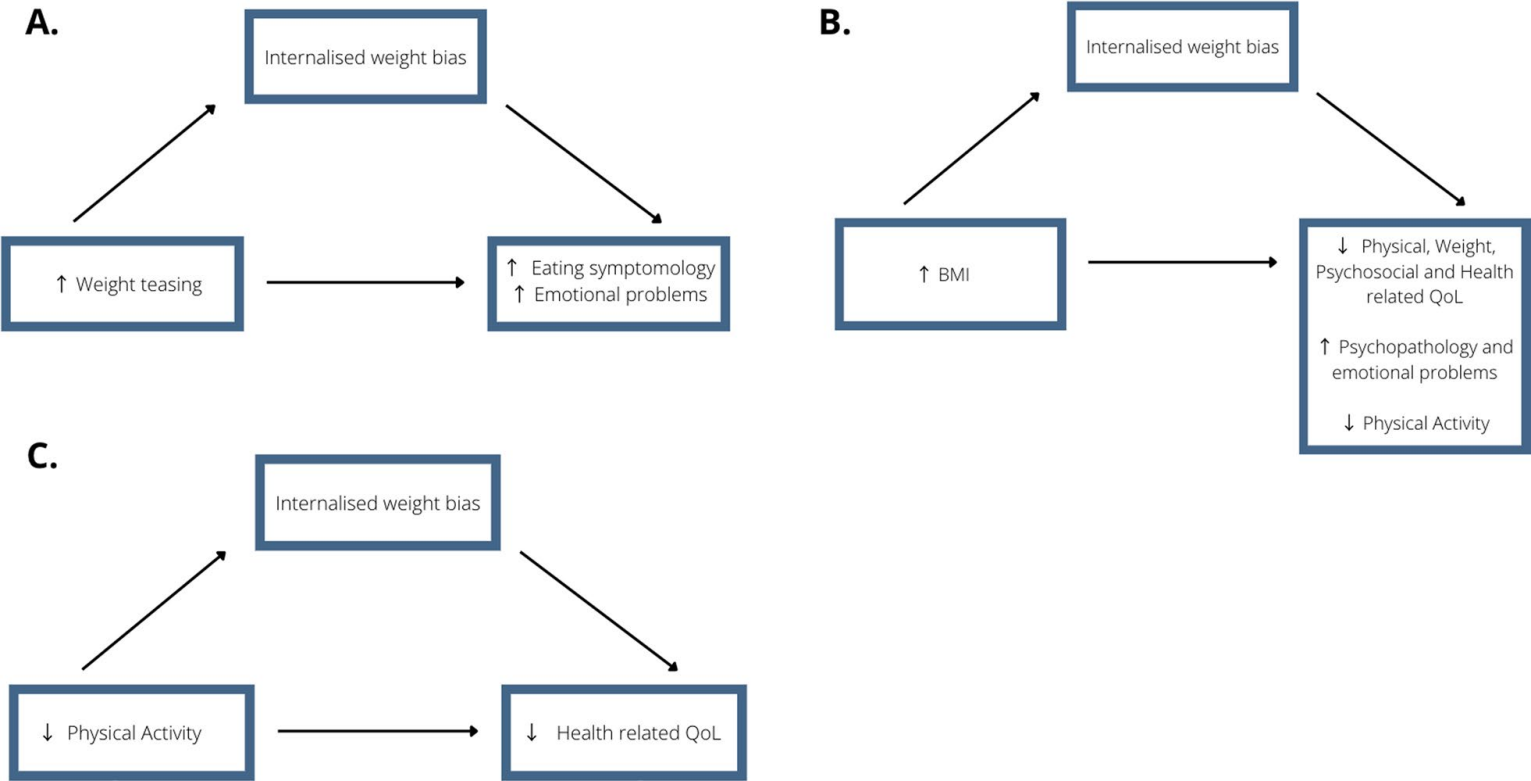
The suggested mediational relationships between IWB and biopsychosocial outcomes. **A** Increased BMI and decreased physical [42], weight-related [49], health-related [39], and psychosocial QoL[42], increased BMI and increased psychopathology [42] and emotional problems [33] and increased BMI and decrease physical activity levels [47]. **B** Increased weight teasing and increased eating symptomology [24, 33] and emotional problems [33]. **C** Decreased physical activity and decreased health related QoL

Fourteen studies investigated associations between BMI and IWB. There was heterogeneity in the data for how BMI was measured. Six articles supplied a mean and standard deviation, whilst six recorded the percentage of weight categories (e.g., ‘underweight’, ‘overweight’), and seven reported both. Fifteen (78.95%) studies relied on self-reported data for weight. Six articles only investigated OW or children and youth with a BMI ≥ 30 kg/m^2^ [21, 36, 41, 42, 45, 46]. Two of the nine studies that explored the weight spectrum had 'normal'/'underweight' participants as a minority, with four of these studies combining the ‘underweight’ and ‘normal’ weight groups [35, 44, 46, 47]. Nine articles found a positive association between IWB and BMI [21, 24, 32–36, 38, 46]. ‘Overweight’ compared to ‘non-overweight’ groups showed higher levels of IWB [24, 36, 39, 44], eating disorder symptomology [24], and decreased quality of life [36, 44], with IWB demonstrating a mediational relationship between physical weight and quality of life [39], Fig. 4.

Two studies [26, 47] investigated the association between IWB and physical activity engagement, finding a statistically significant negative correlation, with IWB scores being lower in active groups when compared to non-active groups.

### Social outcomes

Ten studies investigated social outcomes. Five studies [36, 38, 40, 44] found a positive association between IWB and negative social characteristics in individuals (e.g. conduct issues, attachment to teachers, avoidance behaviours, maladaptive behaviours). One study [32] found that higher IWB scores and lower SES were positively associated. Lastly, eight studies found experiences of experienced weight stigma to have a strong positive association with IWB [24, 32, 33, 37–39, 41, 44].

The findings of this literature are summarised in Table 1 with the demonstrated correlations between IWB. Figure 3 depicts the association between biopsychosocial outcomes and IWB as IWB scores increased. Further mediational relationships can be found in Fig. 4.

### Quality assessment

The quality assessment found three studies to be neutral, and 16 to be positive, indicating good quality of data. However, there was a lack of robust data due to the observational nature of included articles.

## Discussion

This systematic review synthesises the evidence on associations between IWB and biopsychosocial health outcomes in individuals ≤ 25 years. To our knowledge, this review is the first conducted about IWB in individuals ≤ 25 years and confirms that this topic has received limited attention, with a lack of consistency in methods across studies. Despite this lack of coherence, clear associations have been demonstrated between IWB and higher severity of psychological distress, eating disorder symptomology, reduced motivation/self-efficacy to engage in health-promoting behaviours, (e.g., physical activity), higher BMI and decreased positive sociability. Additionally, experiences of weight stigma demonstrated one of the strongest positive associations with IWB, with IWB scores increasing as experiences of weight stigma increased.

### Tools for measuring IWB

This review highlights the need to adopt more objective measures of IWB to enhance confidence in study findings and reduce bias. Eighteen of the 19 studies utilised two self-reporting tools (WBIS and WSSQ) to assess IWB. Although both tools have been shown to demonstrate good psychometric properties and validity, have been translated into several languages, and demonstrate good internal consistency [21], their reliance on self-reported data leaves room for misinterpretation and reporting bias. Furthermore, studies that utilised the WBIS and WSSQ were inconsistent in how they modified tools creating variability in how the scale was used and interpreted. A systematic review of IWB [10] with adults presents the question of how well these tools measure internalisation of weight stigma. For example, the WSSQ measures anticipated stigma from others, which has been differentiated from internalised stigma [10]. Other studies suggested the link between IWB and psychological factors could be attributed to some questions overlapping with other self-judgement factors such as self-esteem and body image [50].

Further, caution should be taken when studying sensitive topics with children, a vulnerable population, to ensure distress is not created for participants. Ciupitu-Plath., et al., found that 4 out of 10 interviewees’ feedback highlighted potential stress that may be caused to participants when filling out the modified WBIS tool. Paediatric healthcare providers also believe that the engagement in weight talk with children and youth patients may cause harm such as increased body dissatisfaction, unhealthy weight control behaviours and negatively impacting patient-provider relationships [51].

Therefore, to improve the quality of future studies, enhance the accuracy when testing the associations between IWB and health outcomes, and ensure such topics are approached in a safe and sensitive way, a tool specifically designed for children and youth is needed. The results of this review highlights three key components of weight internalisation that should be considered; 1) the lived experience and awareness of negative stereotypes 2) the acceptance and agreement of said stereotypes and 3) applying said beliefs to oneself and proceeding to mistreat or devalue oneself as a result [3]. Additionally, it will allow for a higher quality synthesis of findings for future systematic reviews and meta-analyses [21].

### Psychological outcomes

This review identified that IWB held significant positive associations with multiple adverse psychological outcomes. It also identified the need for additional research across the weight spectrum, with studies finding that moderate to strong correlations between IWB and negative psychological health outcomes (e.g. eating disorder symptomology) persisted even after controlling for BMI [12, 52] and held true across weight categories [33]. Pakpour et al., found IWB to be more predictive of psychosocial disturbance and eating disorder symptomology than physical weight [46]. This may be due to the fact physical weight does not take into consideration the complex psychosocial complexities of self-worth/image. Hence, IWB may be more appropriate to screen for in a clinical settings to identify children and youth at risk of developing adverse outcomes rather than BMI alone [46]. However, additional research is required to support these associations using specialised tools.

Of note, preliminary clinical trial studies in adult populations found psychotherapy approaches to be effective in reducing IWB [53, 54], whilst others found that the theory of planned behaviours, health action process approach, and cognitive behavioural therapy were potential techniques to reduce self-stigma [55]. However, none have been trialled in a younger population.

Our review found IWB to have a role as a mediator in the relationship between weight stigma experiences, disordered eating behaviours [24] and psychosocial problems [33] which held true across weight categories. This agrees with findings from studies conducted with adult populations [50]. Our findings suggest that it may be how children and youth intrinsically manage their experiences, beliefs, and physical weight that leads to the development of harmful outcomes through IWB rather than physical weight alone.

### Physical outcomes

Despite the above findings, it is important to recognise that many studies found strong positive associations between increased BMI and IWB levels [21, 24, 32–36, 38, 46]. Many only found this relationship to be true when comparing between groups with a BMI ≥ 25 kg/m^2^ to groups with a BMI < 25 kg/m^2^ [32, 34, 35, 44, 46] but not within populations with a BMI ≥ 25 kg/m^2^ [36, 45]. This is in agreement with previous adult population studies that found no association between current BMI and IWB within populations with a BMI ≥ 25 kg/m^2^ [10]. This may indicate that despite strong associations between higher BMI and IWB scores, the negative influence of IWB reaches a threshold beyond a particular weight. Hence, the extent of higher weight does not explain additional variance in IWB suggesting that individuals who have a higher BMI may internalise weight bias independent of physical weight [12, 36].

Currently, there is a lack of evidence to support that a decrease in BMI will alleviate IWB and its associated negative outcomes. Similarly, while the relationship between negative outcomes and IWB is clear, there is a lack of evidence to support that improving IWB will improve outcomes. However, this review has identified a need for further investigation into children and youth-specific treatment methods. Intervention and cause-effect experimental studies should be utilised with additional research into how IWB can be incorporated into health care practices. Future research should also aid health care professionals to recognise signs of IWB to help identify at-risk children and youth, allowing for early intervention and for the potential prevention of adverse health outcomes that have the tendency to endure into adulthood.

### Strengths and limitations

Strengths of this study include following an explicit methodology with strict eligibility criteria and detailed search strategy to ensure specificity of the topic and allow for transparency and reproduction. It is also the first to investigate the association between IWB and health outcomes in a young population, however, it is not without limitations. Only one reviewer completed the quality assessment indicating potential for reporter bias. Additionally, for the purpose of this paper and due to the heterogeneity of the data, all eating disorder symptomologies were categorised under the same category. This does not take into consideration the complex multifactorial nature of different eating disorder symptomologies or their transdiagnostic properties [56]. Finally, due to the lack of robust data, a meta-analysis was unable to be completed.

### Future direction

This review identified several gaps in current research. This research has contributed to understanding the groundwork for further research into IWB in children and youth to aid in the early identification and prevention of adverse health outcomes prior to them compounding into adulthood, decreasing the risk of lifelong adversities [57].

Firstly, most research was conducted with youth populations, with minimal studies undertaken with children. This could be due to a dearth of validated tools for children, ethics involved in conducting research with children and that they may not be advanced enough cognitively to understand the concepts of internalised weight bias. Children require separate investigation from youth due to fundamental biological, social and hormonal changes occurring during the development into adolescence, which limit the generalisability of our findings [29].

Secondly, a lack of racial and ethnic diversity was evident, with 12 articles not reporting any data on the ethnicity of participants. All studies within western populations were conducted on predominantly white populations. Studies that were conducted on different ethnicities (Middle Eastern and Asian) were unable to be generalised across countries due to cultural differences with the research not adequately capturing the experiences of varying populations. As child development models are not universal but are socially and culturally specific [58], generalisability between nations is difficult. Future research should include more diverse samples in assessments of IWB as it relates to health. Sex-related pathways additionally require further consideration given conflicting evidence on the role that gender contributes to IWB [35]. Finally, in comparison to psychological outcomes, physical and social outcomes were studied considerably less. Greater research should be conducted on these areas using objective measures [59].

Lastly, the cross-sectional nature of these studies do not allow for the formation of a cause-and-effect relationship. More prospective studies are required to investigate the bidirectional relationship between IWB and biopsychosocial outcomes and determine causality. Current research postulates that adverse outcomes may not only be consequences of IWB but also predisposing factors [8, 32], demonstrating a reciprocal predictive relationship, with bidirectional compounding occurring.

Changes to IWB move beyond the individual alone and span to the persons direct environment, family friends and society more broadly. It is however, recognised that changes at a societal level require time, and research such as this review can contribute to our understanding of the relationships in this area. Hence, this research contributes greater understanding to help provide the groundwork for further research into IWB in children and youth to aid in the early identification and prevention of adverse health outcomes prior to them compounding into adulthood, decreasing the risk of lifelong adversities [57].

## Conclusion

This study aimed to identify associations between IWB and adverse health outcomes to inform health professionals of the importance of screening for IWB alongside BMI to identify a youth and children at risk of developing negative health outcomes as a result of IWB. IWB may be more clinically relevant in assessing risk in a population under 25 than physical weight alone due to its psychosocial aspects and ability to transcend beyond BMI. Research would benefit from better assessment tools designed for children and youth populations that accurately measure IWB. Psychotherapies may be an appropriate strategy in addressing IWB given success in adult trials, but research is required for younger populations. This review has contributed to the growing body of literature to help guide future interventions around identifying and comprehending IWB in children and youth.

### Supplementary Information

**Additional file 1:** PRISMA reporting checklist.

## Data Availability

Datasets can be accessed via direct contact to corresponding author.
